# Characterizing the Interactions of Dimethyl Sulfoxide with Water: A Rotational Spectroscopy Study

**DOI:** 10.1021/acs.jpca.2c04599

**Published:** 2022-09-23

**Authors:** Dingding Lv, Luca Evangelisti, Assimo Maris, Wentao Song, Giovanna Salvitti, Sonia Melandri

**Affiliations:** Dipartimento di Chimica “G. Ciamician”, Università di Bologna, via F. Selmi 2, 40126, Bologna, Italy

## Abstract

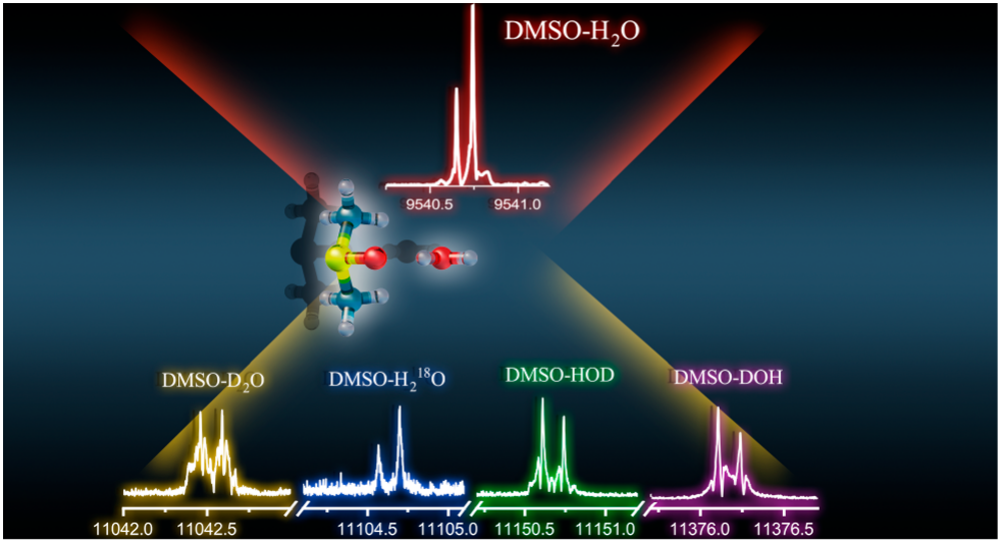

The interaction of dimethyl sulfoxide with water has been investigated by Fourier-transform microwave spectroscopy of the 1:1 complex and its isotopologues, complemented with quantum chemical calculations. The rotational spectra of ^34^S and ^13^C isotopologues in natural abundance and the H_2_^18^O and deuterated water enriched isotopologues have been measured, allowing a partial structure determination and establishing the position of water in the complex. In the most stable conformation water was found to be the donor of a primary OH···OS bond to the oxygen atom of dimethyl sulfoxide and acceptor of two weak CH···OH bonds with the methyl hydrogen atoms of dimethyl sulfoxide. From the structural determination confirmed by quantum chemical calculations, the water molecule lies in the symmetry plane of dimethyl sulfoxide, and the complex has an overall *C*_*s*_ symmetry. The experimental findings are supported by atoms in molecules and symmetry-adapted perturbation theories, which allowed for determining the hydrogen bond and intermolecular interaction energies, respectively.

## Introduction

Dimethyl sulfoxide ((CH_3_)_2_SO, DMSO) is an important substance commonly used as a polar aprotic solvent, antifreeze fluid, and cryo-protectant owing to its beneficial properties including low toxicity and environmental compatibility.^1^ The DMSO/water system is well-known for exhibiting a strongly nonideal mixing behavior. The nonideal behavior of the DMSO/water system, reflected in a number of physical properties, such as the density,^2^ excess mixing volume,^3^ viscosity,^4^ and translational and rotational diffusion constants,^5^ has been a very active field of research for many decades. Insights into how DMSO interacts with water are essential in explaining ice-blocking mechanisms and biological toxicity.^6^

Unique attributes of the DMSO/water system have previously been investigated using various methods, such as molecular dynamics (MD) simulations,^7−9^ neutron diffraction,^10,11^ Rydberg electron-transfer spectroscopy,^12^ high-pressure spectroscopic probing,^13^ FTIR spectroscopy,^14,15^ and Raman spectroscopy.^16^ Kirchner et al. described the stable conformations of DMSO-(water)_*n*_ (*n* = 1, 2, 3, and 4) complexes by MD simulations and calculated the interaction energies of the hydrogen bond (HB) in the stable conformations with different methods.^17^ Mrázková and Hobza studied the conformations of DMSO-(water)_*n*_ (*n* = 1, 3, 6, 16, and 32) using MD simulations and reoptimized DMSO-(water)_*n*_ (*n* = 1, 2, and 3) at the *ab initio* level. They found that the hydration of DMSO leads to an elongation of the S=O bond and a contraction of methyl C–H bonds.^9^ Li et al. studied the cooperativity between O–H···O=S HB and C–H···O_w_ HB in the DMSO aqueous solutions by FTIR spectroscopy and conformations DMSO-(water)_*n*_ (*n* = 1, 2, and 3) by theoretical calculations.^14^ Oh et al. proved singly hydrogen-bonded conformations (DMSO-water and (DMSO)_2_-water) and a doubly hydrogen-bonded conformation (DMSO-(water)_2_) in DMSO/water binary mixture solutions using FTIR spectroscopy combined with theoretical calculations.^15^ They reported that DMSO disrupts the donor/acceptor balance in water by accepting up to two HBs through its oxygen lone pairs. However, despite this extensive research, HB formation preferences in the complex of DMSO with water are not completely understood.

A bottom-up approach to the problem of solvation can be achieved by the study of small molecular clusters by high resolution spectroscopy, in particular rotational spectroscopy,^18^ but also electronic spectroscopy^19,20^ performed in the gas phase. Rotational spectroscopy in particular is highly sensitive to atomic mass distribution, so it can be used to study conformational equilibria and isotopic species. The combination of rotational spectroscopy analysis and theoretical calculations provides a synergistic method for studying the structure and internal dynamics of isolated molecules^21^ and weakly bound complexes.^22^

Herein, we investigate the rotational spectrum of the 1:1 complex of DMSO with water (DMSO-W) and its isotopologues by rotational spectroscopy in the 6–18 GHz region supported by quantum chemical calculations with the aim of determining its structure and the driving forces of the interaction between DMSO and water.

## Methods

The measurement of the rotational spectrum was carried out in a COBRA-type^23^ pulsed jet Fourier-transform microwave (PJ-FTMW) spectrometer^24^ previously described.^25,26^ The sample of DMSO was acquired from Sigma-Aldrich (purity > 99%), while those of H_2_^18^O and D_2_O were purchased from Cambridge Isotopes Inc. (purity > 99.9%) and used without further purification. Samples of DMSO and water were prepared in two separate containers and cooled to 273 K. Helium at a stagnation pressure of about 0.3 MPa was flowed over the samples, resulting in about a 1% mixture of both DMSO and water. The molecular beam was then expanded through a solenoid valve (General Valve, Series 9, nozzle diameter of 0.5 mm) into a Fabry–Pérot cavity. During the expansion, the molecules and their complexes can reach quite low rotational and vibrational temperatures (a few degrees K and less than 100 K, respectively), and the most stable forms can be trapped at their energy minimum when certain conditions are satisfied. The spectral line positions were determined after Fourier transformation of the time-domain signal with 8000 data points, recorded at a sampling interval of 100 ns. The analysis of the spectral data was performed with the SPFIT program of Pickett.^27^

The theoretical search for the stable geometries was performed with the CREST (conformer-rotamer ensemble sampling tool) software,^28,29^ an efficient scheme by the meta-dynamics algorithm combined with semiempirical tight-binding methods. The resulting conformers were optimized using MP2/aug-cc-pVTZ basis sets with the Gaussian 16 program.^30^ The noncovalent interactions between DMSO and water were analyzed with Johnson’s NCI method,^31^ which can visualize and quantify the noncovalent interactions based on a reduced gradient of the electronic density (RDG), atoms in molecules,^32^ and symmetry-adapted perturbation theories.^33^

## Results and Discussion

For the DMSO molecule, extensive research has already been done using rotational spectroscopy performed in the gas phase.^34−39^ The dipole moment and *r*_0_ structure,^38^*r*_s_ structure,^35^ centrifugal distortion constants,^34,37^ and the methyl internal rotation barrier *V*_3_ of DMSO^36^ were previously determined. In terms of chemical structure, the molecule has idealized *C*_*s*_ symmetry and a trigonal pyramidal molecular geometry.

**Figure 1:**
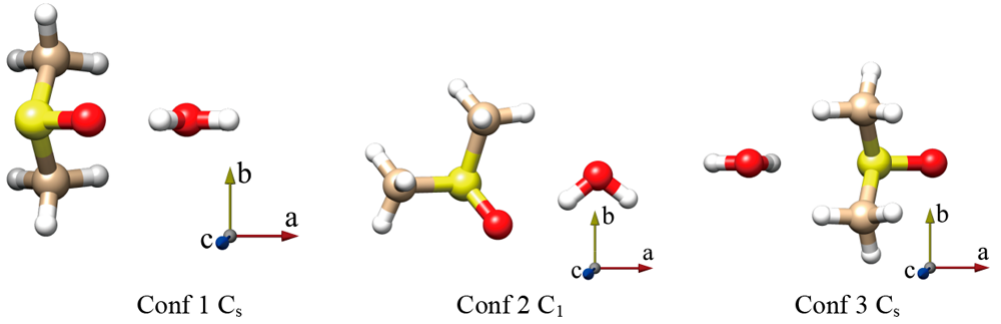
Sketch and principal axis of the three conformers of DMSO-W.

The possible structures of the 1:1 complexes of DMSO with water were predicted using the CREST software^28,29^ obtaining seven nonequivalent species. All of them were optimized at the MP2/aug-cc-pVTZ level of theory obtaining only three stable conformers, as shown in Figure 1. All three conformers were confirmed to be local minima by performing harmonic vibrational calculations. Distinct conformers of DMSO-W have been labeled Conf1, Conf2, or Conf3 based on their increasing relative energy. The theoretical spectroscopic parameters and relative energies are reported in Table 1, while the Cartesian coordinates of the optimized structures are available in the Supporting Information.

**Table 1:** Theoretical (MP2/aug-cc-pVTZ) Spectroscopic Parameters and Relative Energies of the Three Conformers of DMSO-W

	Conf1	Conf2	Conf3
*A* [MHz]	4261	6785	5172
*B* [MHz]	2479	2072	2075
*C* [MHz]	2459	1739	1904
*|μ*_a_| [D]	1.5	3.2	4.1
*|μ*_b|_ [D]	0.0	1.6	0.0
*|μ*_c_| [D]	2.3	0.2	0.9
*P*_aa_ [u Å^2^]^a^	145.4	230.0	205.6
*P*_bb_ [u Å^2^]	60.1	60.6	59.8
*P*_cc_ [u Å^2^]	58.5	13.9	37.9
Δ*E* [kJ mol^–1^]	0.0	6.9	25.1

a The planar moments of inertia *P*_gg_ = Σ_i_*m*_i_*g*_i_^2^ (*g* = *a*, *b*, *c*)

In the most stable conformer (Conf1), the hydrogen of water forms a OH···OS HB with the DMSO oxygen atom, and the water oxygen lone pairs participate in two CH···OH secondary interactions, involving two methyl groups. The structure has a *C*_*s*_ symmetry where the plane of symmetry corresponds to the *ac* inertial plane with the water and the SO group located on it, thus the value of the electric dipole moment component μ_b_ is null. Conformer 2 (Conf2) is located 6.9 kJ mol^–1^ higher than Conf1. In this case, the water molecule lies in the heavy atom plane of DMSO, forming a six-membered ring-like structure where the water hydrogen forms a HB with the DMSO oxygen, and the oxygen atom of water is involved in a HB with a DMSO hydrogen atom. The two lower energy structures exhibit a stronger OH···OS hydrogen bond and one (Conf2) or two (Conf1) weak CH···OS HBs. The hydroxyl group of water is acting as a proton donor for the OH···OS interaction and as a proton acceptor for the CH···OH interaction. In conformer 3 (Conf3), the water lies again in the DMSO symmetry plane and exhibits a OH···SO and two CH···OH interactions. This structure is located 25.1 kJ mol^–1^ higher than Conf1.

We started our experiment searching for the rotational transitions of Conf1. The initial spectral scan has covered a frequency range where the *J*: 2 ←1 μ_a_-type *R* transitions were expected to be found. Using this prediction, some intense rotational transitions have been assigned. Following the rotational pattern, many other μ_a_-type transitions have been recorded and later some μ_c_-type transitions for a total of 17 pure rotational transitions. The lack of μ_b_-type lines confirms the assignment of Conf1 to a structure with an *ac* plane of symmetry.

**Figure 2:**
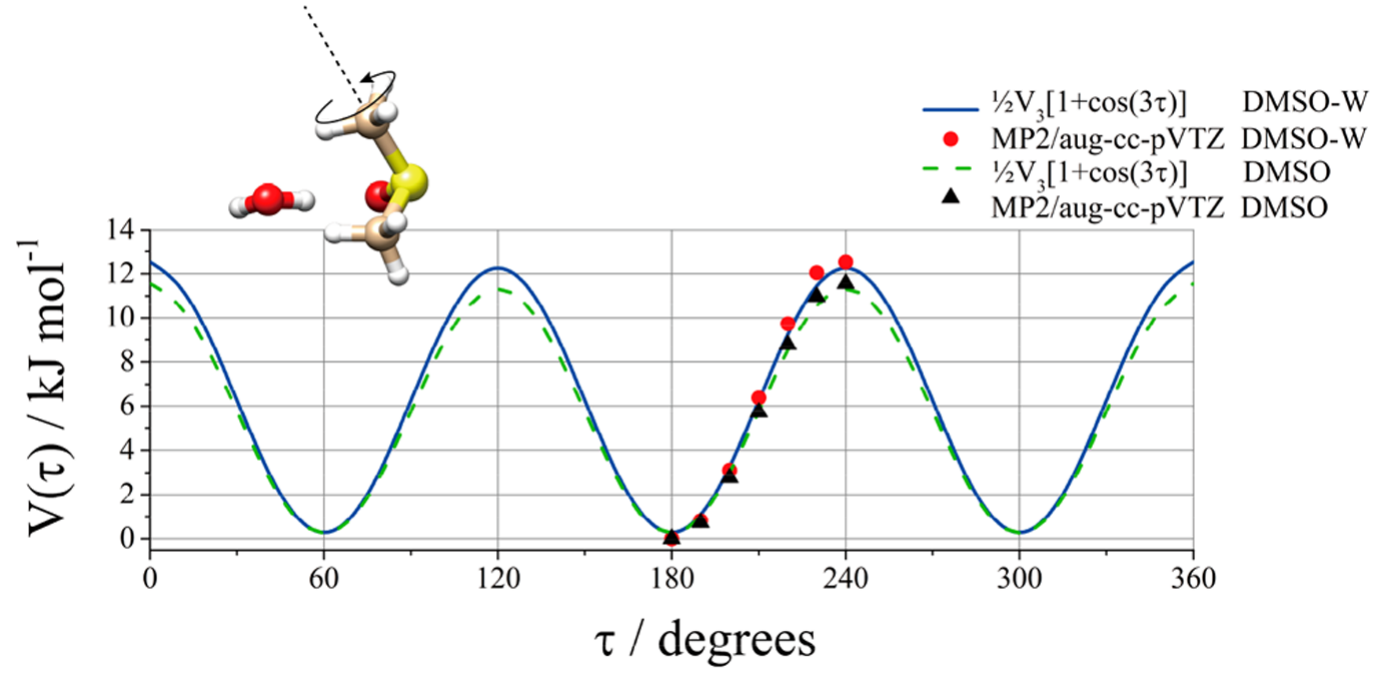
*Ab initio* (MP2/aug-cc-pVTZ) and fitted potential energy surface for methyl internal rotational of DMSO and DMSO-W (Conf1).

In the DMSO monomer, due to the hindered internal rotation of its two equivalent methyl rotors and the *C*_*s*_ symmetry frame, an effective triplet spectral splitting pattern labeled AA, AE (or EA), and EE occurs where A and E are the high-barrier methyl torsional state symmetry labels.^39,40^ Nevertheless, in the case of the observed transitions of Conf1 in our frequency range, no splitting was observed. To verify the different behavior between monomer and complex, the potential energy surface of the methyl torsion around the CS bond in both DMSO and Conf1 was calculated by changing the dihedral angle τ = HCSC in steps of Δτ = 10°, while all of the other parameters were freely optimized. The obtained potential energy curves are compared in Figure 2. The calculated data reported in black triangles and red circles, respectively, are well reproduced with the 3-fold function, *V*(τ) = 1/2*V*_3_[1 + cos(3τ)], which is shown as a green line for DMSO and a blue line for Conf1 of DMSO-W. The maximum value (11.5 kJ mol^–1^ for DMSO and 12.5 kJ mol^–1^ for Conf1) represents the theoretical barrier hindering the methyl group internal rotation, not accounting for zero-point energy contribution. It can be noted that the value of Conf1 is larger (about 12.3%) than the value obtained for DMSO. The increased barrier to methyl rotation in the complex might be related to the interactions between the water oxygen and DMSO hydrogens, and it causes the splitting of the rotational transitions to be below the resolving power of our instrument as they were already very small in the monomer.^39^ The calculated barrier for DMSO is smaller than the experimentally determined one (*V*_3_ = 12.3(3) kJ mol^–1^);^39^ nevertheless, we acknowledge the consistency of the increased value of the internal rotation barrier in going from monomer to complex and the non-observation of the internal rotation splitting in the spectra of the complex.

**Figure 3:**
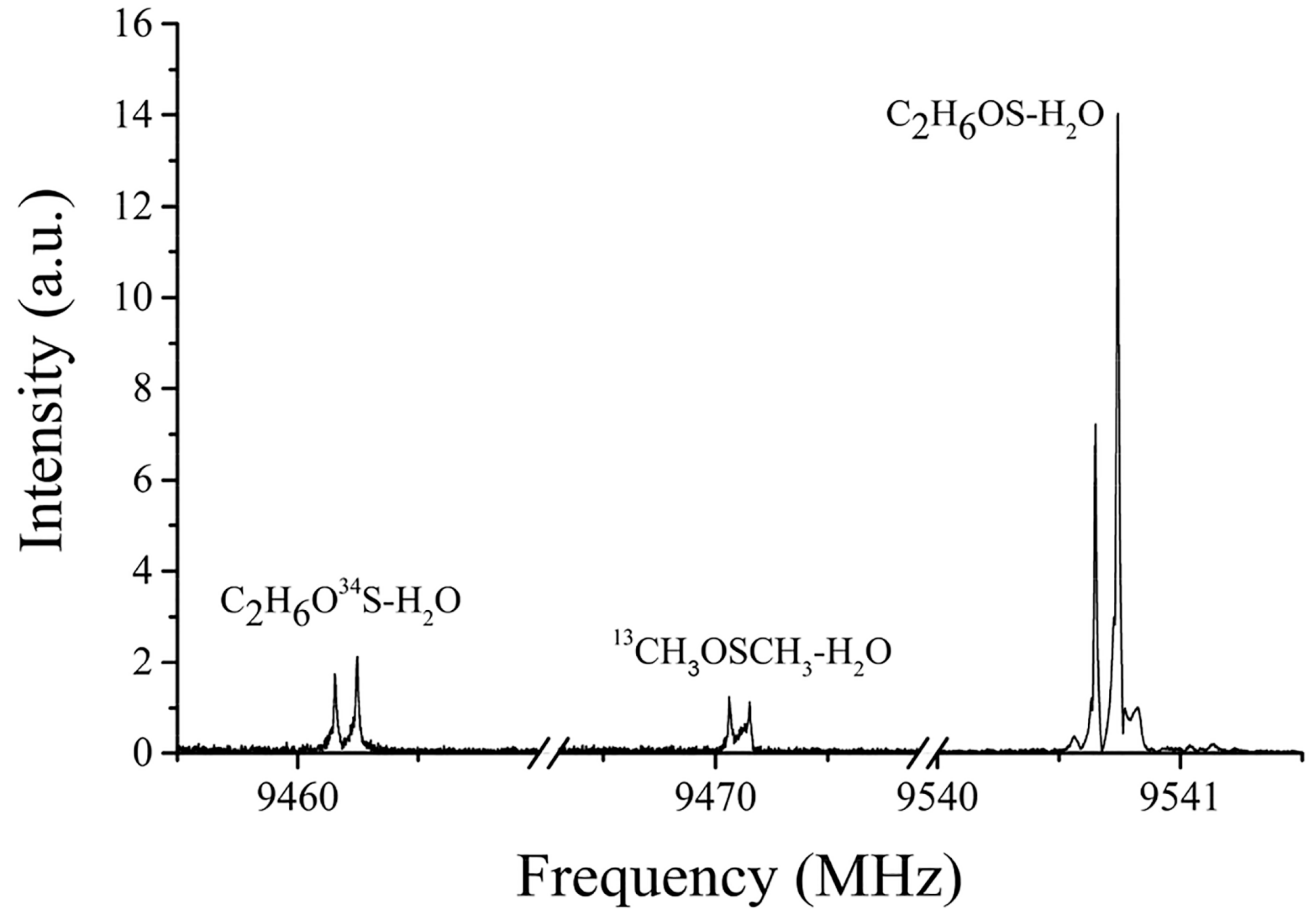
2_0,2_ ← 1_0,1_ transition of parent DMSO-W and ^34^S and ^13^C isotopologues observed in natural abundance. The transition intensity of parent species is 50 au. Each line appears as a doublet due to the instrumental Doppler effect.

Trial rotational constants for the isotopologues were estimated considering their theoretical values and the differences between the experimental and theoretical rotational constants of the parent species. Using this approach, the rotational spectrum of the isotopologues ^34^S and ^13^C were easily identified, measured, and analyzed in natural abundance (4.2% and 2%, respectively). As an example of the observations, the 2_0,2_ ← 1_0,1_ transition for the parent species, ^34^S and ^13^C isotopologues, is shown in Figure 3 where the relative intensity in natural abundance can be inferred.

**Figure 4:**
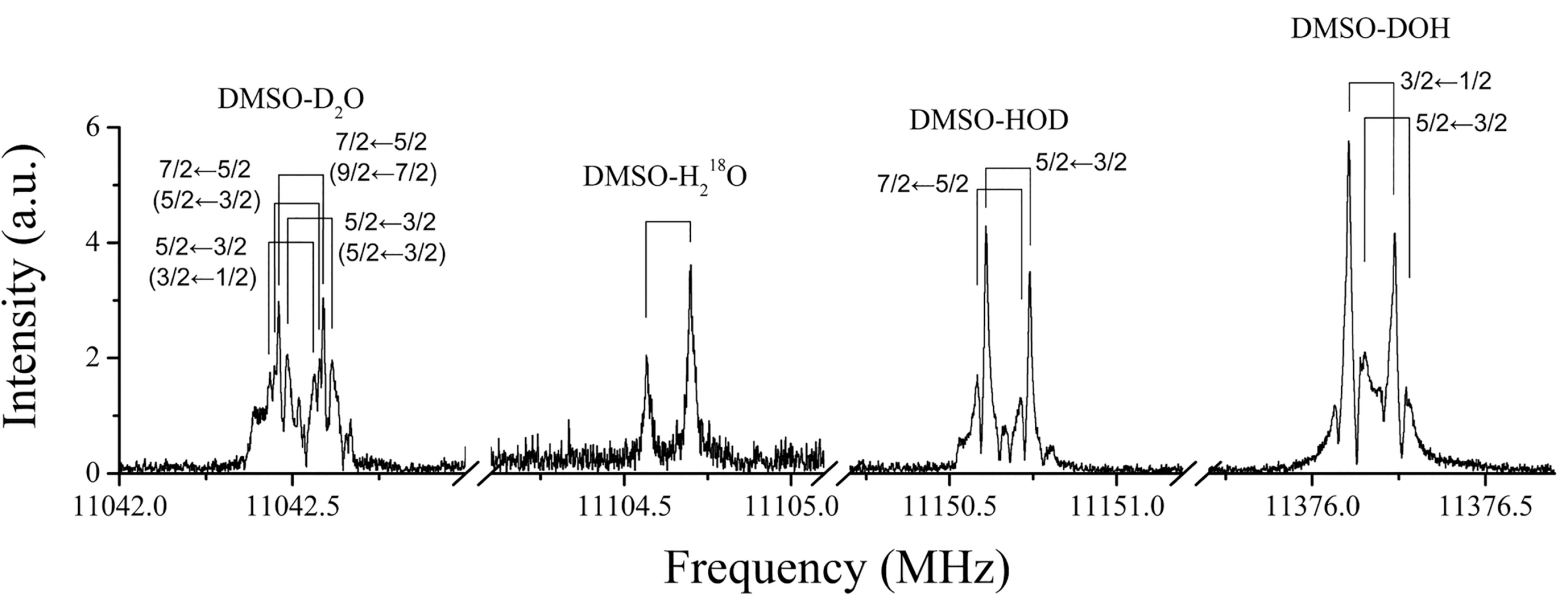
2_1,1_ ← 1_0,1_ transitions of enriched H_2_^18^O and three enriched deuterated species of DMSO-W.

Using ^18^O and deuterium enriched water samples in the experiment, we could also observe the spectra of DMSO-H_2_^18^O and three water deuterated isotopologues. As an example, in Figure 4, we show the corresponding 2_1,1_ ← 1_0,1_ transition for the observed water isotopologues.

The rotational constants of all observed species are summarized in Table 2, while the observed transition frequencies are provided in the Supporting Information (Tables S1–S4). A splitting pattern caused by quadrupole coupling was observed in the spectrum of the deuterated species due to the coupling of the deuterium nuclear quadrupole moment to the overall rotation, as shown in Figure 4. Considering that the splitting pattern is very narrow, some values of the quadrupole coupling constants for the deuterated species (DOH and D_2_O) in Table 2 were fixed to the predicted ones.

**Table 2:** Experimental Spectroscopic Parameters of DMSO-W (Conf1)

fitted	parent	^34^S	^13^C	H_2_^18^O	DOH	HOD	D_2_O
*A* [MHz]	4282.940(4)^a^	4276.711(5)	4194.447(5)	4277.763(4)	4275.679(4)	4280.106(3)	4272.832(2)
*B* [MHz]	2405.027(1)	2383.597(2)	2396.269(2)	2275.665(9)	2366.876(1)	2290.2285(7)	2256.6064(6)
*C* [MHz]	2365.748(1)	2346.902(1)	2339.577(1)	2241.907(1)	2330.8156(9)	2254.6339(6)	2223.8261(5)
*D*_J_ [kHz]	2.79(4)	2.7(5)	2.68(5)	2.59(4)	2.66(4)	2.50(2)	2.40(2)
*D*_JK_ [kHz]	8.4(5)	7.8(8)	8.8(8)	8.1(2)	7.1(4)	7.4(3)	7.8(2)
*D*_K_ [kHz]	–10.3(7)	–9(1)	–10(1)	–9.9(8)	7.7(8)	–8.6(6)	–9.5(5)
3/2χ_aa_ [MHz]					[0.0828]^b^	0.36(1)	[0.0828], [0.36]^c^
(χ_bb_ – χ_cc_)/4 [MHz]					–0.050(3)	–0.024(3)	[−0.050], [−0.024]
σ^d^ [kHz]	1.60	1.54	3.64	2.14	5.8	7.2	8.6
*N*^e^	15	11	11	16	27	37	51
Derived							
*P*_aa_ [u Å^2^]	152.8798(2)	154.5962(2)	153.2139(2)	164.6813(2)	156.0741(1)	163.3712(1)	166.4673(1)
*P*_bb_ [u Å^2^]	60.7435(2)	60.7426(2)	62.7991(2)	60.7425(2)	60.7508(1)	60.7799(1)	60.7893(1)
*P*_cc_ [u Å^2^]	57.2546(2)	57.4275(2)	57.6886(2)	57.3985(2)	57.4477(1)	57.2963(1)	57.4880(1)
χ_aa_ [MHz]					[0.055]^b^	0.245(9)	
χ_bb_ [MHz]					–0.127(9)	–0.170(9)	
χ_cc_ [MHz]					0.072(9)	–0.075(9)	

a Error in parentheses in units of the last digit.

b The data in brackets were fixed to the predicted values.

c The two values correspond to the quadrupole coupling constants for the hydrogen bonded and free deuterium atoms, respectively.

d Standard deviation of the fit.

e Number of lines in the fit.

From the rotational constants of the observed isotopologues, it has been possible to determine the *r*_s_ substitution coordinates using Kraitchman’s equations.^41^ They are compared to the *ab initio* equilibrium coordinates (*r*_e_) in Table 3. Since the atom coordinates in Kraitchman’s equations appear as their squares, their signs cannot be unambiguously determined but must be compared to the calculated ones.^42^ Using this procedure, the *b* substitution coordinates for the water hydrogen atoms are not equal to zero (as predicted by the theoretical calculations) but have a very small value. This can be ascribed to large amplitude motions involving such atoms that are not taken into account in the calculations, since the latter describe the equilibrium structure while the experiment measures the rotational constants in the vibrational ground state. The experimental value of the planar inertia moment along the *b* axis (*P*_*bb*_ = ∑*i*=1^*N*^*m*_*i*_*b*_*i*_^2^) for the observed conformer is *P*_*bb*_ = 60.7435(2)uÅ^2^, which is consistent with the corresponding value of the monomer, *P*_*aa*_ = 60.5498(1) uÅ,^2,31^ confirming that the water molecule and the SO group lie in the symmetry plane of DMSO (the *ac* plane). The constant value of *P*_*bb*_ upon isotopic substitution of these atoms confirms this statement. In the case of the water hydrogen atoms, the previously cited large amplitude vibrations also cause the larger deviation of *P*_*bb*_. The results are in agreement with the MP2/aug-cc-pVTZ calculation for the structure of the complex, and the determined values of the rotational constants are actually reproduced by this theoretical method within 4%.

**Table 3:** Experimental Substitution Coordinates (*r*_s_) and Theoretical MP2/aug-cc-pVTZ Equilibrium Coordinates (*r*_e_) of DMSO-W

	C	S	O_water_	H_water,free_	H_water,HB_
|*a*_s_| [Å]	0.5722(1)^a^	0.93612(4)	2.44993(1)	3.24446(2)	1.78910(3)
|*b*_s_| [Å]	1.34878(6)	*i* 0.022(2)^b^	*i* 0.024(1)	0.2005(3)	0.0845(7)
|*c*_s_| [Å]	0.8348(1)	–0.2999(1)	0.2869(1)	0.2167(3)	0.4481(1)
*a*_e_ [Å]	0.5523	0.9337	–2.3888	–3.2643	–1.7625
*b*_e_ [Å]	–1.3425	0.0000	0.0000	0.0000	0.0000
*c*_e_ [Å]	0.8471	–0.2922	0.2848	–0.1106	–0.4690

a Error in parentheses in units of the last digit.

b In a few cases, the square of the coordinate has been determined to be small and negative, leading to an imaginary value of the coordinate.

**Figure 5:**
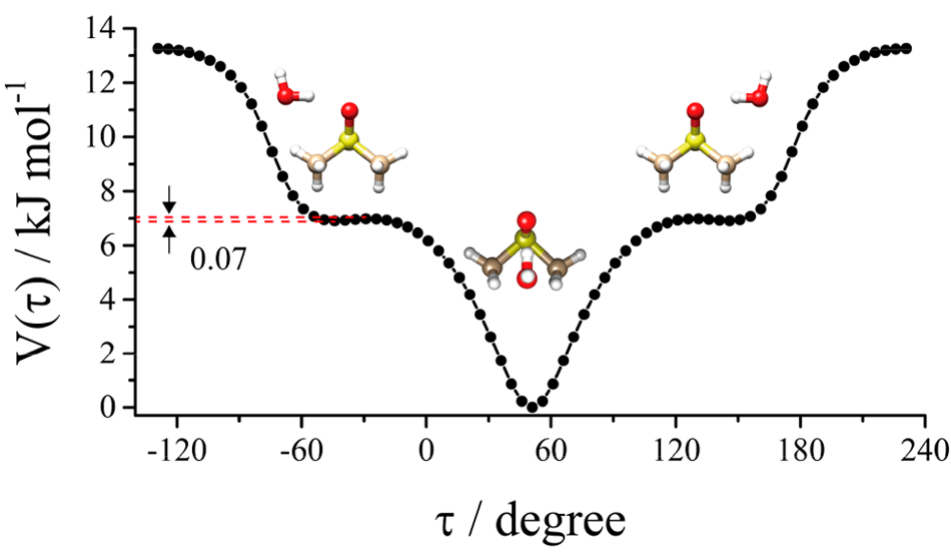
*Ab initio* (MP2/aug-cc-pVTZ) relaxed potential energy surface for the torsion of the water around the S=O axis. This motion interconverts Conf1 (absolute minimum) with Conf2 (two relative equivalent minima).

After measuring all possible transitions of Conf1 (reported in Table S1), the recorded spectrum was extended to search for the rotational transitions of Conf2. Taking into consideration the relative energy, the values of the dipole moment components and the temperature prior to the expansion, the most intense μ_a_ lines of Conf2 were predicted to be 26% of the most intense ones of Conf1, which should make them observable given the overall intensity of the spectrum. However, the rotational transitions of Conf2 were not observed, and this can be attributed to a possible relaxation of the population of this conformation onto that of the global minimum during adiabatic expansion. In order to confirm this, the relaxed potential energy surface was calculated for the torsion of the water molecule around the S=O axis in steps of 5°, as shown in Figure 5. The global minimum corresponds to Conf1, and the two relative equivalent minima correspond to Conf2. The extremely small interconversion barrier between Conf2 and Conf1 suggests that the lack of observation is likely due to a relaxation process of the higher energy form onto the global minimum^43^ or to the absence of any vibrational state inside the potential energy well.

**Figure 6:**
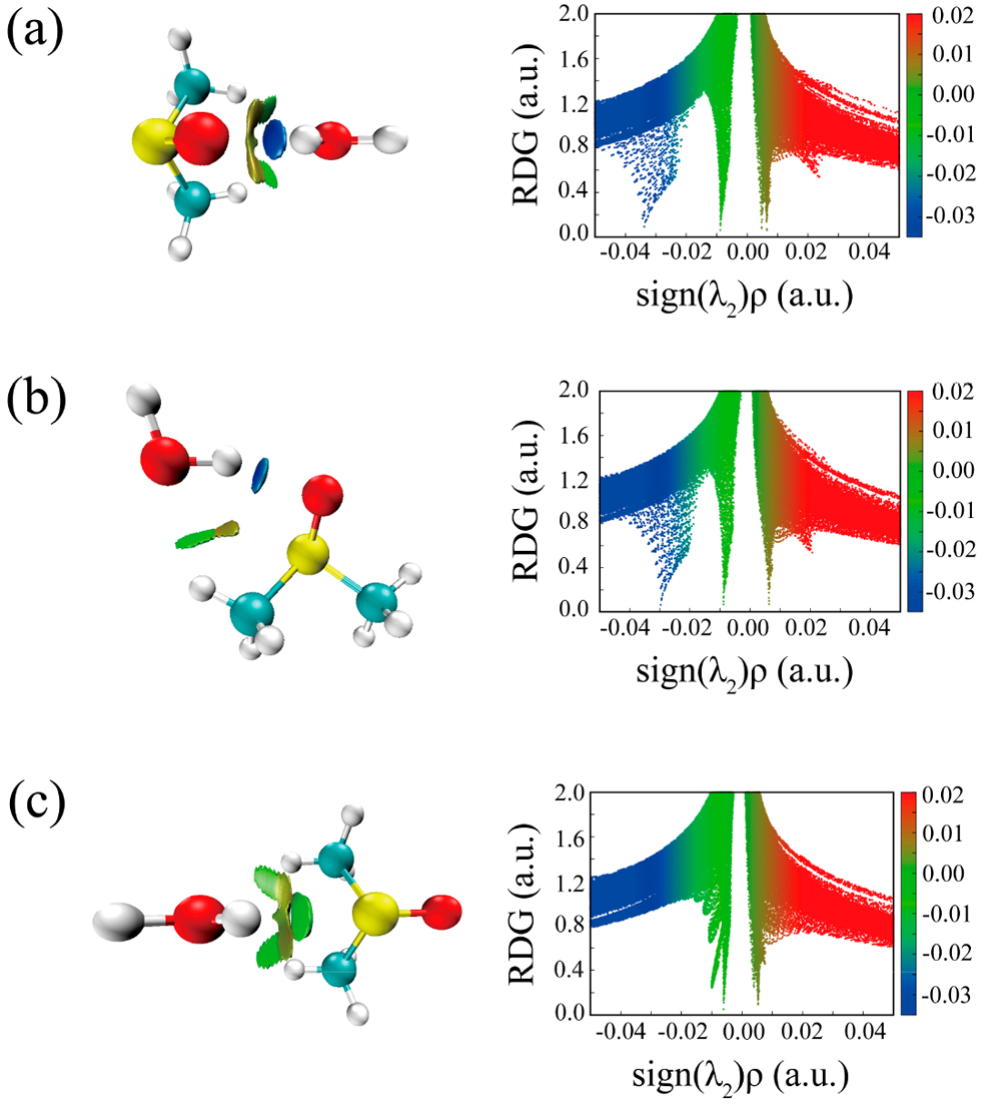
NCI plots from the *ab initio* (MP2/aug-cc-pVTZ) outputs for three conformers: (a) Conf1, (b) Conf2, and (c) Conf3. Left panel: gradient isosurfaces according to the sign(λ_2_)ρ (−0.04–0.04 au). Color coding is blue (strong attractive interactions), green (weak attractive interactions), and orange-red (repulsive interaction). Right panel: The RDG versus sign(λ_2_)ρ. Positive sign(λ_2_)ρ indicates repulsive interactions, and negative sign(λ_2_)ρ indicates attractive interactions.

In order to get a better visualization of the noncovalent interactions between the molecules, the analysis of the HBs in the three stable conformers was performed using Johnson’s NCI method.^31^ The NCI method considers the distribution of the electron density (ρ), its gradient (*s*), and the three eigenvalues (λ_1_, λ_2_, λ_3_) of the electron density Hessian (second derivative) matrix. A comprehensive picture can be drawn using different plots of these quantities. According to the color code reported on the graphics, the gradient isosurfaces (*s* = 0.5 au) visible in the NCI plots represent the area for attractive (negative sign(λ_2_)ρ, blue), repulsive (positive sign(λ_2_)ρ, orange), and weak (green) interactions. The comparison among conformers 1, 2, and 3 is shown in Figure 6a–c. A strong OH···OS HB and two weak CH···OH HBs were observed in Conf1. A similar OH···OS and only one CH···OH HB are seen in Conf2, while three weaker OH···SO and CH···OH interactions are present in Conf3.

**Figure 7:**
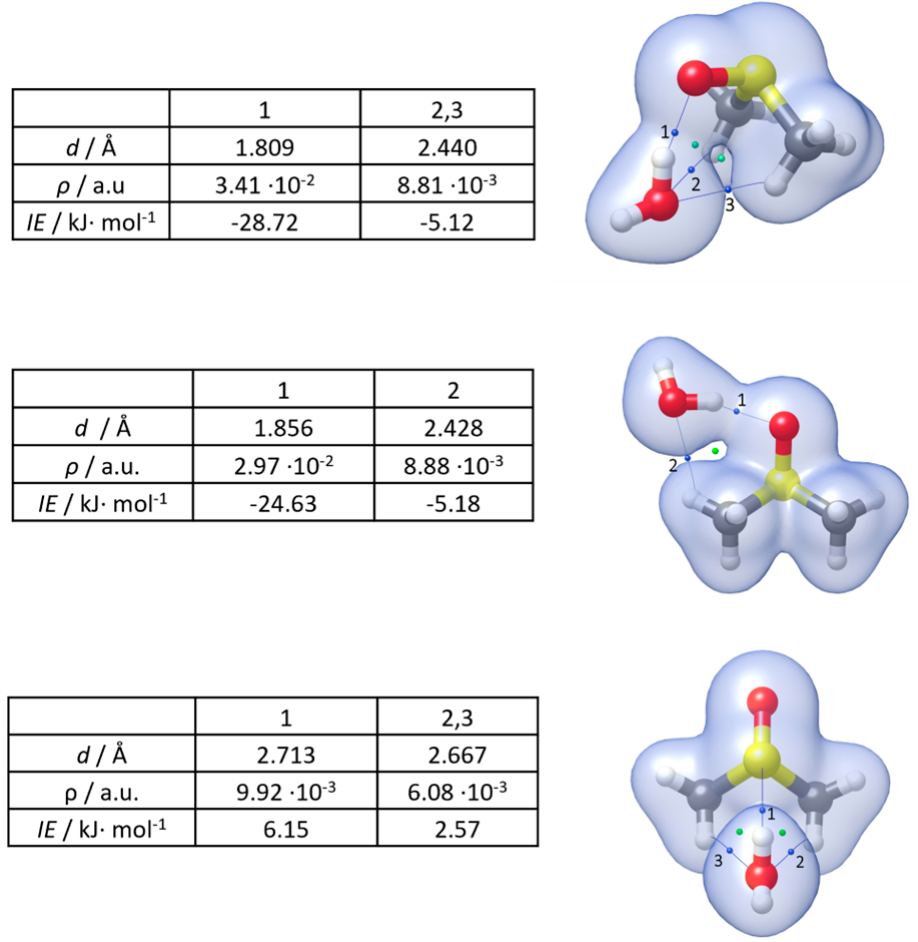
Electron density surface (in blue, isovalue 0.01 au); BCPs and RCPs (in blue and green, respectively) of Conf1, Conf2, and Conf3 of DMSO-W (MP2/aug-cc-pVTZ geometry).

In order to quantify and rank those interactions, a Bader’s Quantum Theory Atoms in Molecules (QTAIM)^44^ analysis has been performed on the MP2/aug-cc-pVTZ wave functions by using the Multiwfn program.^45^ This analysis allows the determination of the bond critical points (BCPs), which are saddle points in the electron density space function that identify the covalent and noncovalent interactions in a molecular or supramolecular system. The BCPs related to the previously visualized interactions have been identified for all conformers. They are reported in Figure 7 together with the corresponding electron density and bond length values. In the same figure, there is evidence of ring-like structure formation characterized by ring critical points (RCP).

The electron density at a certain BCP is intuitively an indication of the strength of the corresponding interaction. In 2019, the following equation to derive the interaction energy (IE) of an HB in neutral complexes from the density at the corresponding BCP was proposed by Emamian and co-workers:^46^
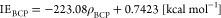
1

The IE_BCP_ for each noncovalent interaction of the investigated complexes is reported in kJ·mol^–1^ in Figure 7, while the sums of the IE_BCP_ are reported in Table 4, where they are compared to the total IEs derived by the application of the supramolecular approach and the symmetry-adapted perturbation theory (SAPT) approach.^33^ By a supramolecular approach, we mean the estimation of the binding energy by subtracting from the energy of the complex those of the isolated monomers. We computed energies at the MP2/aug-cc-pVTZ level in two ways:

**Table 4:** Theoretical Intermolecular Binding and Interaction Energies (kJ·mol^–1^) for DMSO-W

	Conf1	Conf2	Conf3
IE_BCP_	–38.95	–29.81	–11.29
BE_Δ_	–44.43	–37.53	–19.35
IE_Δ_	–45.99	–38.71	–19.67
IE_SAPT_	–44.98	–38.46	–17.34
electrostatic	–74.42	–58.61	–23.12
exchange-repulsion	85.24	63.76	25.70
induction	–27.43	–21.53	–5.07
dispersion	–28.36	–22.08	–14.85

•As the difference between the energy of the binary molecular complex (A–B) and the energy of the two constituting units (A and B) in their minimum configuration
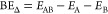
4

•As the difference between the energy of the molecular complex and the energy of the isolated monomers at the geometry within the complex (A* and B*)
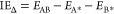
5

SAPT is a perturbative approach used to compute the IE as a perturbation to the Hamiltonian of the individual monomers. It allows not only the estimation of the total IE but also the decomposition of it into electrostatic, exchange, induction, and dispersion components. In this work, we applied a high order SAPT approach (DF-SAPT2+(3)δMP2/aug-cc-pVTZ) implemented in the Psi4 package.^47^ The BE and IE values obtained are reported in Table 4 together with the SAPT contributions.

All methods predicted Conf1 as the highest binding energy species, followed by Conf2 and Conf3, in agreement with the observed conformational preference. By inspecting the results, we can notice that, first, the two supramolecular approaches give similar results, suggesting that the geometry rearrangement contribution to the total BE is negligible and, second, that the supramolecular and SAPT approaches agree, whereas the values derived as the sum of the BCPs’ contributions are smaller. The underestimation can be ascribed both to the nonapplicability of formula 1 to large complexes with multiple and highly nondirectional HBs and to there possibly being more to be taken into account besides the IE at the BCPs.

## Conclusions

The rotational spectrum of the 1:1 DMSO-W has been assigned in the frequency range 6–18 GHz with PJ-FTMW spectroscopy, and the observed conformation determined to have the water molecule is located within the symmetry plane of DMSO (the *ac* plane of the complex). This is proved by the value of the planar inertia moment *P*_bb_, which is consistent with the *P*_aa_ of the DMSO monomer. The hypothesis is also supported by the fact that transitions involving μ_b_ were not observed. The observations of the isotopologues of DMSO (^34^S and ^13^C) in natural abundance and the enriched water isotopologues (H_2_^18^O and deuterated species) allowed the determination of the experimental *r*_s_ coordinates for the S and C atoms of DMSO and the O and H atoms of water. A primary OH···OS HB exists between the water hydrogen and the DMSO oxygen where the water acts as the proton donor and the DMSO as a proton acceptor, and two weak interactions exist between the terminal CH groups and the water oxygen where the DMSO acts as the proton donor and the water as a proton acceptor.

The observed structure of the 1:1 complex shows a very high interaction energy (about 45 kJ mol^–1^) primarily due to the OH···OS HB, which accounts for more than 70% of the total energy mainly attributed to the electrostatic contribution. This high stability of small clusters has been proposed to be responsible for the unusually low melting point observed in DMSO water mixtures.^17^

## Supporting Information Available

Experimental transition frequencies of observed species of DMSO-W, MP2/aug-cc-pVTZ geometries, and atom numbering adopted for DMSO-Water (Conf1) (PDF)
