# Supplementary material for: Characterizing the Interactions of Dimethyl Sulfoxide with Water: A Rotational Spectroscopy Study
Source: J Phys Chem A. 2022 Sep 23;126(39):6882–9. doi: 10.1021/acs.jpca.2c04599 (PMC9549460; doi:10.1021/acs.jpca.2c04599)
Supplement: Supplementary file 1 — jp2c04599_si_001.pdf [file jp2c04599_si_001.pdf]

ELECTRONIC SUPPLEMENTARY MATERIAL

Characterizing the Interactions of Dimethylsulfoxide with Water: a Rotational Spectroscopy Study

Dingding Lv, Luca Evangelisti, Assimo Maris, Wentao Song, Giovanna Salvitti, and Sonia Melandri*

Dipartimento di Chimica "G. Ciamician",Università di Bologna, via F. Selmi 2, 40126, Bologna Italy

E-mail: sonia.melandri@unibo.it

CONTENTS

Table S1. Experimental transition frequencies (ν/MHz) and obs-calc. values ($\Delta\nu$/MHz) of several isotopologues of DMSO-W (Conf1).

Table S2. Experimental transition frequencies (ν/MHz) and obs-calc. values ($\Delta\nu$/MHz) of DMSO-DOH (Conf1).

Table S3. Experimental transition frequencies (ν/MHz) and obs-calc. values ($\Delta\nu$/MHz) of DMSO-HOD (Conf1).

Table S4. Experimental transition frequencies (ν/MHz) and obs-calc. values ($\Delta\nu$/MHz) of DMSO-D_2O (Conf1).

Table S5. MP2/aug-cc-pVTZ geometry of the three conformers of DMSO-W complexes.

Figure S1. Atom numbering adopted for DMSO-Water (Conf1).

Table S1. Experimental transition frequencies (v/MHz) and obs-calc. values (Δv/MHz) of several isotopologues of DMSO-W (Conf1).

J'	K_a'	K_c'	J''	K_a''	K_c''	C_2H_6OS-H_2O		$C_2H_6O^{34}S$-H_2O		$^{13}CH_3OSCH_3$-H_2O		C_2H_6OS-$H_2{}^{18}O$	
						v	Δv	v	Δv	v	Δv	v	Δv
μ_a-lines													
2	1	2	1	1	1	9502.1507	0.0016	9424.1848	-0.0010	9414.8785	-0.0017	9001.2722	0.0015
2	0	2	1	0	1	9540.8510	-0.0006	9460.3826	-0.0010	9470.2885	0.0003	9034.6371	-0.0004
2	1	1	1	1	0	9580.7044	-0.0024	9497.5736	-0.0018	9528.2573	-0.0073	9068.785	-0.0007
3	1	3	2	1	1	14252.6774	-0.0009					13501.4849	-0.0026
3	0	3	2	0	2	14309.5895	0.0027					13550.7418	-0.0009
3	2	2	2	2	1							13552.2444	0.0043
3	2	1	2	2	0							13553.9288	-0.0041
μ_c-lines													
1	1	0	0	0	0	6687.9495	0.0001	6660.2927	0.0020	6590.6998	0.0012	13602.7587	0.0020
2	1	1	1	0	1	11497.8908	-0.0013	11427.3769	-0.0011	11383.1282	0.0011	6553.412	0.0014
2	2	0	1	1	0	15215.0590	0.0006	15177.4460	0.0021	14924.1213	0.0038	11104.6338	-0.0010
2	2	1	1	1	1	15253.7250	-0.0025	15213.6082	-0.0021	14979.4863	-0.0038	15672.7544	0.0004
3	1	2	2	0	2	16327.5519	0.0026	16212.8654	0.0013	16204.2427	0.0023	15075.5084	0.0031
2	0	2	1	1	0	7623.6678	0.0015	7530.5832	0.0022	7615.4325	0.0069	15108.8365	-0.0031
3	0	3	2	1	1	12352.5475	0.0011	12222.1005	0.0003	12289.1360	-0.0023	6998.788	-0.0004
4	0	4	3	1	2	17058.3352	-0.0011	16892.2629	-0.0004	16927.2843	0.0001	11480.7465	0.0011
3	1	3	2	2	1	8501.0993	-0.0005						
3	1	2	2	2	0	8736.1557	-0.0015						

Table S2. Experimental transition frequencies (v/MHz) and obs-calc. values (Δv/MHz) of DMSO-DOH (Conf1).

J'	K_a'	K_c'	$F'+½$	J''	K_a''	K_c''	$F''+½$	v	Δv
μ_a-lines									
2	1	2	1	1	1	1	0	9053.9309	0.014
2	1	2	3	1	1	1	2	9054.0020	-0.004
2	1	2	2	1	1	1	2	9054.0392	0.008
2	1	2	1	1	1	1	1	9054.0642	0.019
2	0	2	3	1	0	1	2	9089.1657	0.000
2	0	2	2	1	0	1	1	9089.1657	-0.005
2	1	1	3	1	1	0	2	9125.1904	-0.003
2	1	1	2	1	1	0	1	9125.2605	-0.010
2	1	1	2	1	1	0	2	9125.2605	0.011
3	1	3	2	2	1	2	1	13580.5730	-0.005
3	1	3	4	2	1	2	3	13580.5915	0.010
3	1	3	3	2	1	2	2	13580.5915	-0.010
3	0	3	3	2	0	2	2	13632.4211	-0.003
3	0	3	4	2	0	2	3	13632.4211	-0.001
3	1	2	4	2	1	1	3	13687.3591	-0.001
3	1	2	2	2	1	1	1	13687.3591	-0.004
3	1	2	3	2	1	1	2	13687.3812	-0.001
4	1	4	5	3	1	3	4	18106.6089	-0.014
4	0	4	5	3	0	3	4	18174.0849	0.004
4	1	3	5	3	1	2	4	18248.9933	0.015
μ_c-lines									
1	1	0	1	0	0	0	1	6570.3000	0.000
1	1	0	2	0	0	0	1	6570.3258	0.004
2	1	1	2	1	0	1	1	11150.6476	-0.009
2	1	1	3	1	0	1	2	11150.6742	-0.001
3	1	2	4	2	0	2	3	15748.8595	-0.009

3	1	2	3	2	0	2	2	15748.8697	0.001
3	1	2	2	2	0	2	1	15748.8916	0.005
2	2	0	3	1	1	0	2	15095.3266	0.002
2	2	1	2	1	1	1	1	15130.4100	-0.003
2	2	1	3	1	1	1	2	15130.4419	0.001
2	0	2	1	1	1	0	1	7063.6600	-0.003
2	0	2	3	1	1	0	2	7063.6793	-0.005
3	0	3	4	2	1	1	3	11570.9096	-0.003
3	0	3	3	2	1	1	2	11570.9421	0.003
4	0	4	5	3	1	2	4	16057.6381	0.004
4	0	4	4	3	1	2	3	16057.6381	0.000
4	0	4	3	3	1	2	2	16057.6381	-0.001

Table S3. Experimental transition frequencies (ν/MHz) and obs-calc. values ($\Delta\nu$/MHz) of DMSO-HOD (Conf1).

J'	K_a'	K_c'	$F'+½$	J''	K_a''	K_c''	$F''+½$	ν	$\Delta\nu$
μ_a-lines									
2	1	2	1	1	1	1	0	9359.1684	0.005
2	1	2	2	1	1	1	2	9359.1876	0.003
2	1	2	3	1	1	1	2	9359.2073	0.000
2	0	2	3	1	0	1	2	9394.7940	0.003
2	0	2	2	1	0	1	1	9394.7940	0.002
2	1	1	1	1	1	0	1	9431.2871	0.007
2	1	1	3	1	1	0	2	9431.3212	-0.002
2	1	1	2	1	1	0	1	9431.3409	-0.002
3	1	3	4	2	1	2	3	14038.3347	-0.004
3	0	3	3	2	0	2	2	14090.7686	0.006
3	0	3	4	2	0	2	3	14090.7686	0.006
3	0	3	2	2	0	2	1	14090.7686	0.002
3	1	2	4	2	1	1	3	14146.5246	0.011
3	1	2	3	2	1	1	2	14146.5246	0.006
3	1	2	2	2	1	1	1	14146.5246	0.005
μ_c-lines									
1	1	0	2	0	0	0	1	6642.5303	-0.003
1	1	0	1	0	0	0	1	6642.5612	0.005
2	1	1	3	1	0	1	2	11376.1715	-0.007
2	1	1	2	1	0	1	1	11376.2058	0.001
3	1	2	4	2	0	2	3	16127.8931	-0.008
3	1	2	3	2	0	2	2	16127.9341	0.002
2	2	0	3	1	1	0	2	15158.2368	-0.002
2	2	1	3	1	1	1	2	15193.7761	-0.007
2	2	1	2	1	1	1	1	15193.8102	0.005
2	2	1	1	1	1	1	1	15193.8365	0.004
3	0	3	4	2	1	1	3	12109.3596	-0.013
4	0	4	5	3	1	2	4	16747.8925	0.003

Table S4. Experimental transition frequencies (ν/MHz) and obs-calc. values ($\Delta\nu$/MHz) of DMSO-D_2O (Conf1).

J'	K_a'	K_c'	$F'+½$	$F''+½$	J''	K_a''	K_c''	$(F'+½)$	$(F''+½)$	ν	$\Delta\nu$
2	1	2	3	3	1	1	1	2	2	8927.9521	-0.009

2	1	2	3	4	1	1	1	2	3	8927.9688	0.007
2	1	2	2	3	1	1	1	2	2	8928.0103	0.004
2	1	2	3	2	1	1	1	1	1	8928.0221	0.000
2	1	2	2	3	1	1	1	1	2	8928.0555	0.003
2	0	2	3	3	1	0	1	2	2	8960.3832	-0.005
2	1	1	3	2	1	1	0	0	1	8993.4460	-0.012
2	1	1	1	2	1	1	0	2	1	8993.4751	-0.011
2	1	1	3	4	1	1	0	2	3	8993.5085	-0.007
2	1	1	1	2	1	1	0	1	1	8993.5466	0.017
2	1	1	3	2	1	1	0	1	1	8993.5589	0.015
2	1	1	3	3	1	1	0	1	2	8993.5672	0.001
2	1	1	2	3	1	1	0	2	3	8993.5825	0.001
3	1	3	3	3	2	1	2	2	2	13391.5705	-0.007
3	1	3	3	4	2	1	2	2	3	13391.5918	0.002
3	0	3	4	5	2	0	2	3	4	13439.4541	0.005
3	1	2	4	5	2	1	1	3	4	13489.8981	-0.003
4	1	4	5	6	3	1	3	4	5	17854.7029	0.000
4	0	4	5	6	3	0	3	4	5	17917.1494	-0.001
4	1	3	5	6	3	1	2	4	5	17985.8070	0.002
4	1	3	5	5	3	1	2	4	4	17985.8111	0.001
1	1	0	1	1	0	0	0	1	2	6529.3689	-0.011
1	1	0	1	2	0	0	0	1	1	6529.4019	-0.002
1	1	0	2	3	0	0	0	1	2	6529.4230	0.000
1	1	0	2	2	0	0	0	1	2	6529.4500	0.008
1	1	0	0	1	0	0	0	1	1	6529.4779	0.012
2	1	1	2	1	1	0	1	1	0	11042.4993	-0.001
2	1	1	2	3	1	0	1	1	2	11042.5143	-0.003
2	1	1	3	4	1	0	1	2	3	11042.5269	-0.003
2	1	1	3	3	1	0	1	2	2	11042.5493	0.000
2	1	1	1	0	1	0	1	0	1	11042.5861	0.000
2	1	1	2	3	1	0	1	2	3	11042.6002	0.004
2	2	0	2	3	1	1	0	1	2	15042.5608	-0.012
2	2	0	3	3	1	1	0	2	2	15042.5928	-0.008
2	2	0	3	2	1	1	0	2	2	15042.6045	-0.017
2	2	0	3	4	1	1	0	2	3	15042.6421	0.007
2	2	0	1	2	1	1	0	2	1	15042.6826	0.006
2	2	0	3	2	1	1	0	1	1	15042.7004	0.017
2	2	0	1	2	1	1	0	1	2	15042.7102	0.014
2	2	1	2	3	1	1	1	2	2	15074.9281	-0.016
2	2	1	1	2	1	1	1	0	1	15074.9496	-0.011
2	2	1	2	3	1	1	1	1	2	15074.9827	-0.007
2	2	1	3	4	1	1	1	2	3	15075.0038	0.000
2	2	1	3	3	1	1	1	2	2	15075.0177	0.007
2	2	1	3	3	1	1	1	1	2	15075.0738	0.017
2	0	2	3	4	1	1	0	2	3	6911.3736	0.003
3	0	3	4	4	2	1	1	3	3	11357.2806	-0.007
3	0	3	4	5	2	1	1	3	4	11357.3039	0.000
3	0	3	3	4	2	1	1	2	3	11357.3233	0.002
4	0	4	5	5	3	1	2	4	4	15784.5207	-0.006
4	0	4	5	6	3	1	2	4	5	15784.5593	0.007

Table S5. MP2/aug-cc-pVTZ geometry of the three conformers of DMSO-W complexes

	a[Å]	*b*[Å]	*c*[Å]			*a*[Å]	*b*[Å]	*c*[Å]			*a*[Å]	*b*[Å]	*c*[Å]
Conf1					Conf2					Conf3			
C	-0.55231	-1.34245	-0.84710		C	-0.06956	1.50345	0.16179		C	-0.13266	-1.33931	0.84419
H	-0.68099	-2.26866	-0.29219		H	0.95278	1.64490	-0.18165		H	-0.34076	-2.26772	0.31759
H	-1.25157	-1.30395	-1.68128		H	-0.72668	2.27447	-0.23819		H	0.91157	-1.29753	1.14855
H	0.47995	-1.23035	-1.17573		H	-0.09928	1.47503	1.25007		H	-0.81223	-1.22463	1.68749
C	-0.55228	1.34263	-0.84681		C	-2.19056	-0.13166	0.38954		C	-0.13173	1.33853	0.84494
H	-0.68094	2.26872	-0.29169		H	-2.65050	-1.09057	0.16422		H	-0.33927	2.26737	0.31888
H	0.47998	1.23061	-1.17547		H	-2.00355	-0.04231	1.45844		H	-0.81132	1.22382	1.68822
H	-1.25154	1.30433	-1.68099		H	-2.81294	0.68277	0.02169		H	0.91250	1.29593	1.14920
S	-0.93373	0.00000	0.29219		S	-0.60439	-0.09881	-0.45898		S	-0.45535	0.00000	-0.31708
O	0.17266	0.00000	1.33188		O	0.24864	-1.13273	0.25124		O	-1.93597	0.00000	-0.58700
O	2.38877	0.00000	-0.28484		O	2.76939	0.05663	0.08928		O	2.71268	0.00000	-0.33100
H	3.26428	0.00000	0.11064		H	2.00749	-0.54365	0.19279		H	3.60636	0.00000	-0.68713
H	1.76253	0.00000	0.46896		H	3.51874	-0.52119	-0.07565		H	2.13978	0.00000	-1.10675

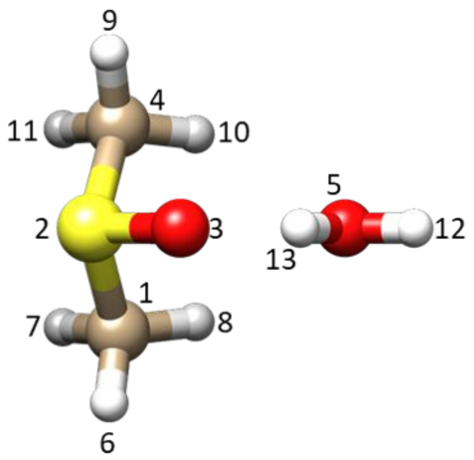

Figure S1. Atom numbering adopted for DMSO-Water (Conf1).
